# Antimicrobial Activity of Poly-epsilon-lysine Peptide Hydrogels Against *Pseudomonas aeruginosa*

**DOI:** 10.1167/iovs.61.10.18

**Published:** 2020-08-10

**Authors:** Stephnie M. Kennedy, Pallavi Deshpande, Andrew G. Gallagher, Malcolm J. Horsburgh, Heather E. Allison, Stephen B. Kaye, Donald A. Wellings, Rachel L. Williams

**Affiliations:** 1 Department of Eye and Vision Science, Institute of Life Course and Medical Sciences, University of Liverpool, Liverpool, United Kingdom; 2 SpheriTech Ltd, Runcorn, Cheshire, United Kingdom; 3 Department of Infection Biology and Microbiomes, Institute of Infection, Veterinary and Ecological Sciences, University of Liverpool, Liverpool, United Kingdom; 4 Department of Clinical Infection, Microbiology and Immunology, Institute of Infection, Veterinary and Ecological Sciences, University of Liverpool, Liverpool, United Kingdom

**Keywords:** hydrogel, pseudomonas keratitis, contact lenses, antimicrobial, bandage lens

## Abstract

**Purpose:**

To determine the antimicrobial activity of poly-epsilon-lysine (pɛK) functionalization of hydrogels against *Pseudomonas aeruginosa*.

**Methods:**

Antimicrobial activities of pɛK and pɛK+ hydrogels were tested against both keratitis and a laboratory strain of *P* *aeruginosa* at a range of inocula sizes, over 4 and 24 hours. The number of viable CFU on pɛK and pɛK+ hydrogels or commercial contact lenses (CL) was investigated. Ex vivo porcine corneas were inoculated with *P* *aeruginosa* PAO1 (10^3^ CFU) and incubated with pɛK+ hydrogels or commercial hydrogel CL for 24 hours and the effects of infection determined.

**Results:**

PɛK+ hydrogels showed log reductions in viable CFU compared with pɛK hydrogels for all *P* *aeruginosa* strains, depending on inocula sizes and incubation time. After 24 hours pɛK+ hydrogels showed >5 and >7.5 log reduction in CFU compared with commercial hydrogel CL at 10^3^ and 10^6^ CFU, respectively. In an ex vivo porcine corneal infection model, pɛK+ hydrogels led to a significant decrease in viable PAO1 CFU and histologic analysis indicated a decreased infiltration of PAO1 into the stroma.

**Conclusions:**

PɛK+ hydrogels demonstrated enhanced antimicrobial activity versus nonfunctionalized pɛK hydrogels against clinically relevant *P* *aeruginosa* strains. PɛK+ hydrogels have the potential to be used as a bandage CL with innate antimicrobial characteristics to minimize the risk of microbial keratitis.

Microbial keratitis (MK), is a significant cause of vision loss and blindness.^1^^,^^2^ Although the corneal surface maintains a barrier to invading microorganisms,^3^^,^^4^ infection may occur when this barrier is disrupted as a result of contact lens (CL) wear, corneal surgery, trauma, or in ocular surface disease.^5^^–^^7^ *Pseudomonas aeruginosa* is an opportunistic, gram-negative, pathogenic bacterium and is one of the leading causes of MK.^8^^,^^9^ *P* *aeruginosa* MK leads to rapid destruction of the cornea and increased disease severity than many other bacteria. *P* *aeruginosa* keratitis is commonly associated with CL wear,^9^^,^^10^ with the CL itself contributing to the development of infections, owing to hypoxia and trauma.^11^

*P* *aeruginosa* possesses an array of virulence factors; however, only some of them are implicated in MK. Expression of a type III secretion system translocates virulence/effector proteins into host cells. Four effector proteins have been identified and characterized for *P* *aeruginosa*: exotoxins (Exo) U, S, T and Y.^12^ The ExoU and ExoS proteins are not usually both expressed owing to variable gene carriage.^13^^–^^15^ ExoU is cytotoxic and results in lysis of cells,^16^ whereas ExoS is associated with tissue invasion and induces apoptosis.^17^^,^^18^ The *exoU* cytotoxic genotype is more common than *exoS* in MK and associated with greater resistance to antibiotics and CL disinfectant solutions.^19^^–^^21^

Ocular surface disease and corneal surgery can compromise the corneal surface, owing to epithelium damage. These conditions are often treated with topical steroids to decrease inflammation, leaving the eye more susceptible to invading pathogens.^22^ In these circumstances, treatment often incorporates the application of a CL as a bandage to protect the wounded cornea. Current CL have no antimicrobial properties and are often used as a bandage CLs in conjunction with topical broad-spectrum antimicrobials to minimize the risk of infection. The development of antimicrobial CLs that prevent the initial adhesion and colonization of bacteria could decrease the need for the administration of topical antimicrobials and counteract evolving antimicrobial resistance.^23^

Poly-epsilon-lysine (pɛK) is a cationic peptide with intrinsic antimicrobial properties and broad-spectrum antimicrobial activity against gram-positive and -negative bacteria, yeasts, and fungi.^24^^,^^25^ We have previously demonstrated that pɛK hydrogels decreased the growth of laboratory strains of *Staphylococcus aureus* and *Escherichia coli*.^26^ The mechanism of action of pɛK previously described involves disruption of the cell membrane and the cell wall.^27^^,^^28^ Cross-linking pɛK with a dicarboxylic acid using carbodiimide chemistry results in a transparent hydrogel with excellent mechanical properties that can be cast into a CL and is nontoxic to corneal epithelial cells.^26^ PɛK hydrogels, therefore, have the potential to be used as a bandage CL. The antimicrobial activity of pɛK hydrogels can be enhanced by covalently binding additional pɛK to the hydrogels free amine groups (pɛK+), as previously described by Gallagher et al,^26^ which showed approximately a 10-fold increase in amine functionality after functionalization owing to the additional pɛK molecules, compared with the nonfunctionalized hydrogel. In this study, we compared the relative antimicrobial properties of pɛK+ hydrogels with nonfunctionalized pɛK hydrogels and commercial hydrogel CL using *P* *aeruginosa* PAO1 and two different clinical isolates, *exoU^+^* and *exoS^+^,* in both in vitro assays and an ex vivo corneal infection model*.*

## Methods

### PɛK Hydrogel Synthesis and Functionalization

The fabrication of pɛK hydrogels (cross-linked to 60 mol% with octanedioic acid to a polymer density of 0.071 g mL^−1^), pɛK functionalization, sterilization, and wash steps were performed as previously described.^26^^,^^29^

### Bacterial Strains and Culture Conditions

*P* *aeruginosa* (PAO1) (ATCC 47085) and clinical keratitis isolates (cytotoxic strain *exoU^+^* PA39016 and invasive strain *exoS^+^* PA58017) (kindly donated by Prof. C. Winstanley, University of Liverpool, Liverpool, UK),^30^ were cultured overnight on Luria–Bertani (LB) agar (Sigma-Aldrich, Dorset, UK) plates at 37°C. Five milliliters of LB broth (Sigma-Aldrich) was inoculated with bacteria and cultured for 18 hours with shaking (120 rpm/min), at 37°C. Overnight *P* *aeruginosa* cultures were subcultured in LB broth (1 in 100 dilution) until an optical density at 600 nm of 0.5 was achieved, corresponding to 10^8^ CFU mL^−1^ and centrifuged at 3000×*g* for 10 minutes in a prechilled centrifuge (4°C). Pellets were washed in chilled, sterile PBS and recentrifuged as described elsewhere in this article. Pellets were re-suspended into 10 mL PBS and serially diluted to produce separate 100 µL inocula corresponding with 10^3^ to 10^7^ bacteria in 10-fold increments.

### In Vitro Antimicrobial Activity of pɛK Hydrogels Against *P aeruginosa*

Under sterile conditions, 6-mm diameter discs of test materials (pɛK and pɛK+ hydrogels, LB agar discs or commercial hydrophilic cast-molded CL; Hydrogel CL, (Filcon II 2, 77% water content, Ultravision, Leighton Buzzard, UK) were transferred to 96-well plates (Greiner, Stonehouse, UK) using forceps. Inocula were incubated with test materials for 4 or 24 hours at 37°C with shaking (120 rpm). After incubation, plates were transferred to ice to minimize further bacterial growth. Test materials were removed from suspensions to fresh 96-well plates containing 100 µL PBS per well and washed twice with shaking at 120 rpm for 30 seconds, to remove loosely attached bacteria. After washing, test materials were transferred into a further 100 µL fresh PBS buffer and vortexed for 30 seconds to remove adherent bacteria into suspension. Bacteria from the original overnight culture and suspended bacteria were serially diluted and 10 µL of each dilution plated onto LB agar plates using the Miles and Misra method^31^ and incubated at 37°C for 24 hours for enumeration. After vortexing in PBS buffer, pɛK and pɛK+ hydrogels, and LB agar discs were placed directly onto LB agar plates and incubated overnight at 37°C to determine bacterial regrowth. A minimum of four independent experiments were performed for each condition, with a minimum of three repeats for each condition per experiment.

### Ex Vivo Corneal Culture

Fresh porcine eyes were obtained from 6-month-old pigs within 6 hours of slaughter from a local abattoir and corneas were excised as previously described.^32^ Eyes containing visible lacerations identified using 2% (w/v) Fluorescein sodium (Bausch & Lomb, Kingston-upon-Thames, UK) were excluded from the study. Corneas were washed for 2 minutes in sterile PBS containing 1% (v/v) penicillin, streptomycin, and amphotericin B (Sigma Aldrich), followed by a 2-minute wash in 3% (v/v) iodinated povidone (Ecolab Ltd, Leeds, UK) and washed thoroughly in antibiotic-free PBS. Corneas were placed epithelial side down into sterile bijou tube lids. UltraPure Agarose (Thermo Fisher Scientific, Loughborough, UK) (0.5% [w/v]) dissolved in Dulbecco's modified eagles medium (approximately 65.5°C), cooled to approximately 37°C and pipetted onto the endothelial side of corneas to fill the cavity and solidified at room temperature (approximately 25°C). Corneas and agarose supports were transferred into 6-well plates, epithelial side up, containing 3 mL Dulbecco's modified eagles medium (antibiotic free, containing 10% [v/v] fetal bovine serum [Labtech, Heathfield, UK]) and incubated at 37°C in 5% CO_2_ for 24 hours before PAO1 infection, to ensure they were antibiotic and infection free before the start of the assay.^33^

### Ex Vivo Corneal Infection

Corneal epithelia were debrided using sterile 6-mm filter paper discs (Grade AA Discs, Whatman, Maidstone, UK) soaked in 70% (v/v) EtOH placed onto cornea for 5 seconds, followed by removal of epithelium with a surgical blade. Corneas were rinsed in Dulbecco's modified eagles medium and air-dried in a laminar flow cabinet for 10 minutes before inoculation. An inoculum of 10 µL (10^3^ CFU PAO1) was seeded onto the central cornea and incubated at room temperature for 15 minutes, enabling attachment of the PAO1 into the corneal surface, with minimal movement of the cornea. Sterile 8.5-mm diameter pɛK+ hydrogels or commercial hydrogel CLs were placed onto the corneal surface. Control infected and noninfected, de-epithelialized corneas were run in parallel with 10 µL PBS added onto cornea as a mock inoculum, plus control corneas with epithelium intact. Corneas were incubated at 37°C in 5% CO_2_ for 24 hours.

After overnight PAO1 infection, the central infected area of the cornea, or the cornea area under pɛK+ hydrogels or commercial hydrogel CLs, was trephined using a sterile 8.5-mm diameter CORONET long-handled corneal trephine (Network Medical Products Ltd, Ripon, UK), transferred into 500 µL PBS and homogenized using a Qiagen Tissue ruptor (Qiagen, Manchester, UK). Suspended bacteria were serially diluted in PBS and plated onto LB agar plates as previously described and quantified as CFU/cornea. Four independent experiments were performed, with a minimum of three corneas for each condition. A separate set of corneas from each independent experiment were fixed in 10% (v/v) neutral buffered formalin overnight for histology.

### Histology

Corneas were processed using a Leica ASP300 tissue processor. Paraffin-embedded tissue was sectioned at a thickness of 5 µm and stained with a Gram stain (Tissue) kit (Thermofisher, Loughborough, UK) following the manufacturer's protocol and imaged using a Nikon CI upright microscope using a 60× objective.

### Statistical Analysis

Bacterial counts were log_10_ transformed before data analysis. Data are presented as standard deviation of the mean and statistical analyses was carried out using GraphPad Software Prism version 8.4.1 (La Jolla, CA) using a two-way ANOVA and Tukey's post hoc analysis. A *P* value of less than 0.05 was considered significant.

## Results

### Antimicrobial Activity of pɛK+ Hydrogels Against *P aeruginosa*

**Figure 1.**
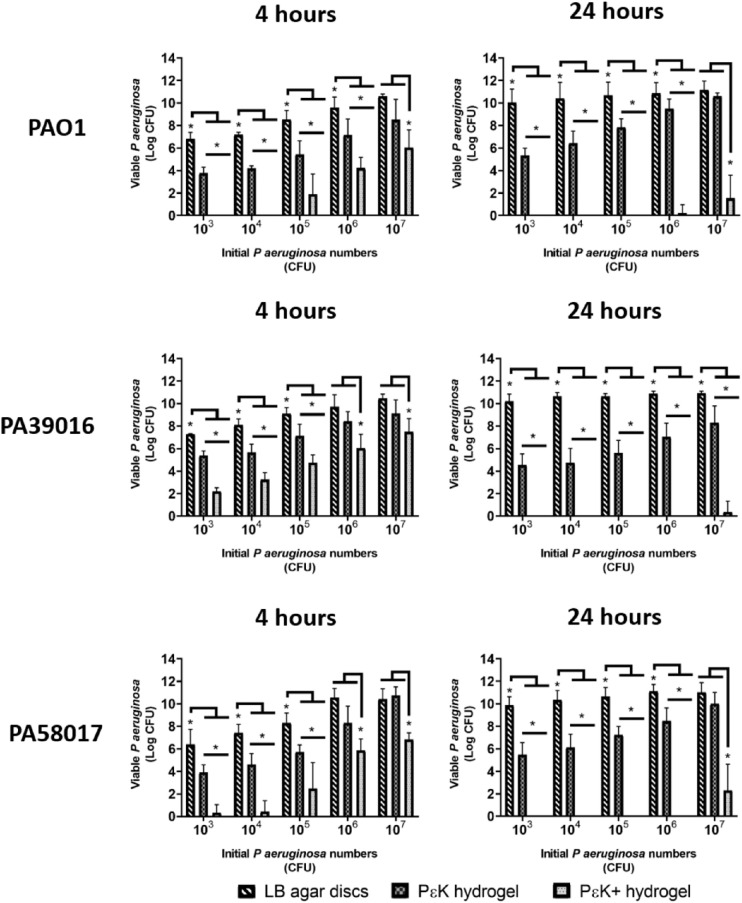
Antimicrobial activity of pԑK+ hydrogel lenses, compared with pԑK hydrogels and LB agar discs against *P* *aeruginosa* isolates in PBS buffer. PԑK+ hydrogel, pԑK hydrogel lenses and LB agar discs were inoculated with *P* *aeruginosa* (PAO1, PA39016, and PA58017) at 10^3^, 10^4^, 10^5^, 10^6^, and 10^7^ CFU for 4 and 24 hours. Viable bacterial counts were determined as CFU from PBS buffer. Values represent mean, error bars represent the standard deviation. *P < 0.05 using two-way ANOVA and post hoc Tukey's analysis.

The innate antimicrobial activity of pɛK+ hydrogels compared with nonfunctionalized pɛK hydrogels was determined against *P* *aeruginosa* PAO1 and two clinically relevant strains (PA39016 [*exoU^+^*] and PA58017 [*exoS*^+^]) at varying inocula sizes (10^3^, 10^4^, 10^5^, 10^6^, and 10^7^ CFU) cultured in PBS buffer. The number of viable CFU from the surrounding PBS buffer of the hydrogels was determined after 4 or 24 hours incubation. LB agar discs served as positive controls for *P* *aeruginosa* bacterial growth. PɛK+ hydrogels showed increased antimicrobial activity compared with nonfunctionalized pɛK hydrogels (Fig. 1; see Supplementary Table S1 for log reductions). Data showed significant decreases in viable CFU within the PBS buffer for all *P* *aeruginosa* strains and inocula after 4 and 24 hours incubation. PAO1 and PA58017 showed decreases between 2 and 4 log, whereas PA39016 showed decreases between 1.63 and 2.43 log, depending on inocula size, after 4 hours incubation. After 24 hours incubation, pɛK+ hydrogels decreased the number of viable CFU further by 4.53 to 9.27 log, depending on the strain and inocula size.

PɛK+ hydrogels decreased the number of viable PAO1 CFU in the PBS buffer after 4 hours incubation to below the starting inocula. PA39016 showed decreases in viable CFU below the starting inocula for 10^3^ and 10^4^ CFU, but no decreases at higher starting inocula (Fig. 1). PA58017 showed decreases in viable CFU below the starting inocula for 10^3^, 10^4^, and 10^5^ CFU, but not at the higher inocula. After 24 hours incubation, viable CFU were below the level of detection in the PBS buffer for starting inocula between 10^3^ and 10^5^ CFU for all *P* *aeruginosa* strains. At the higher starting inocula of 10^6^ and 10^7^ CFU, viable CFU were less than 10^1^ CFU for PAO1 and PA39016 and less than 10^3^ CFU for PA39016. Nonfunctionalized hydrogels showed the numbers of viable CFU greater than the starting inocula at 4 and 24 hours.

### Determination of *P aeruginosa* Associated With pɛK+ Hydrogels

**Figure 2.**
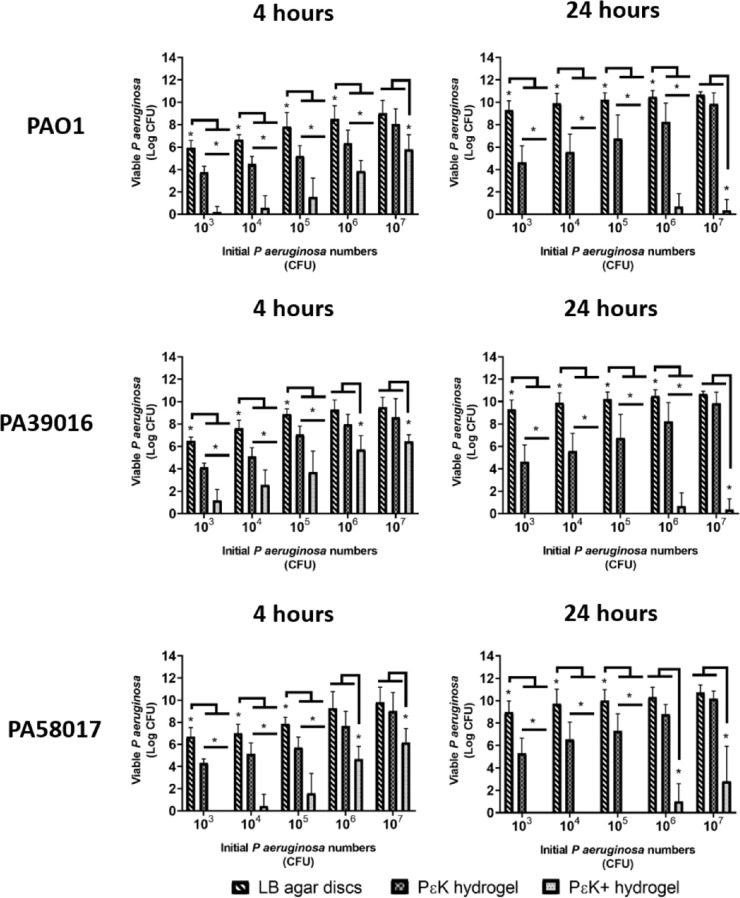
Antimicrobial activity of pԑK+ hydrogels, compared with pԑK hydrogels and LB agar discs against *P* *aeruginosa* isolates. PԑK+ hydrogels, pԑK hydrogel, and LB agar discs were inoculated with *P* *aeruginosa* (PAO1, PA39016, and PA58017) at 10^3^, 10^4^, 10^5^, 10^6^, and 10^7^ CFU for 4 and 24 hours. Viable bacterial counts associated with pԑK+ hydrogels, pɛK hydrogels, or LB agar discs were determined as CFU. Values represent mean, error bars represent the standard deviation. *P < 0.05 using two-way ANOVA and post hoc Tukey's analysis.

We investigated the number of *P* *aeruginosa* that remained associated with the hydrogels after incubation with different *P* *aeruginosa* strains and inocula sizes. *P* *aeruginosa* associated with pɛK+ hydrogels was washed off and resuspended into PBS buffer and viable counts determined for each strain at 4 and 24 hours. Data showed the number of viable PAO1 associated with pɛK+ hydrogels was reduced, compared with nonfunctionalized pɛK hydrogels with inocula from 10^3^, 10^4^, 10^5^, 10^6^, and 10^7^ CFU at 4 hours with corresponding 3.58, 3.90, 3.67, 2.50, and 2.28 log decreases, respectively (Fig. 2A; see Supplementary Table S2 for log reductions). Similar effects in viable count reductions of both strains PA39016 and PA58017 for pɛK+ hydrogels compared with nonfunctionalized pɛK hydrogels with all inocula sizes were determined.

Pronounced differences between the functionalized and nonfunctionalized pɛK hydrogels were determined at 24 hours. PɛK+ hydrogels decreased the number of associated PAO1 CFU compared with nonfunctionalized pɛK hydrogels by 4.75, 5.98, 7.85, 8.76, and 8.42 log, with inocula of 10^3^, 10^4^, 10^5^, 10^6^, and 10^7^ CFU, respectively (Fig. 2; see Supplementary Table S2 for log reductions). To a similar extent, pɛK+ decreased viable CFU compared with nonfunctionalized pɛK hydrogels at inocula of 10^3^, 10^4^, 10^5^, 10^6^, and 10^7^ CFU for both keratitis isolates PA39016 and PA58017 at 24 hours.

### *P aeruginosa* Growth From pɛK+ Hydrogels Onto LB Agar Plates

**Figure 3.**
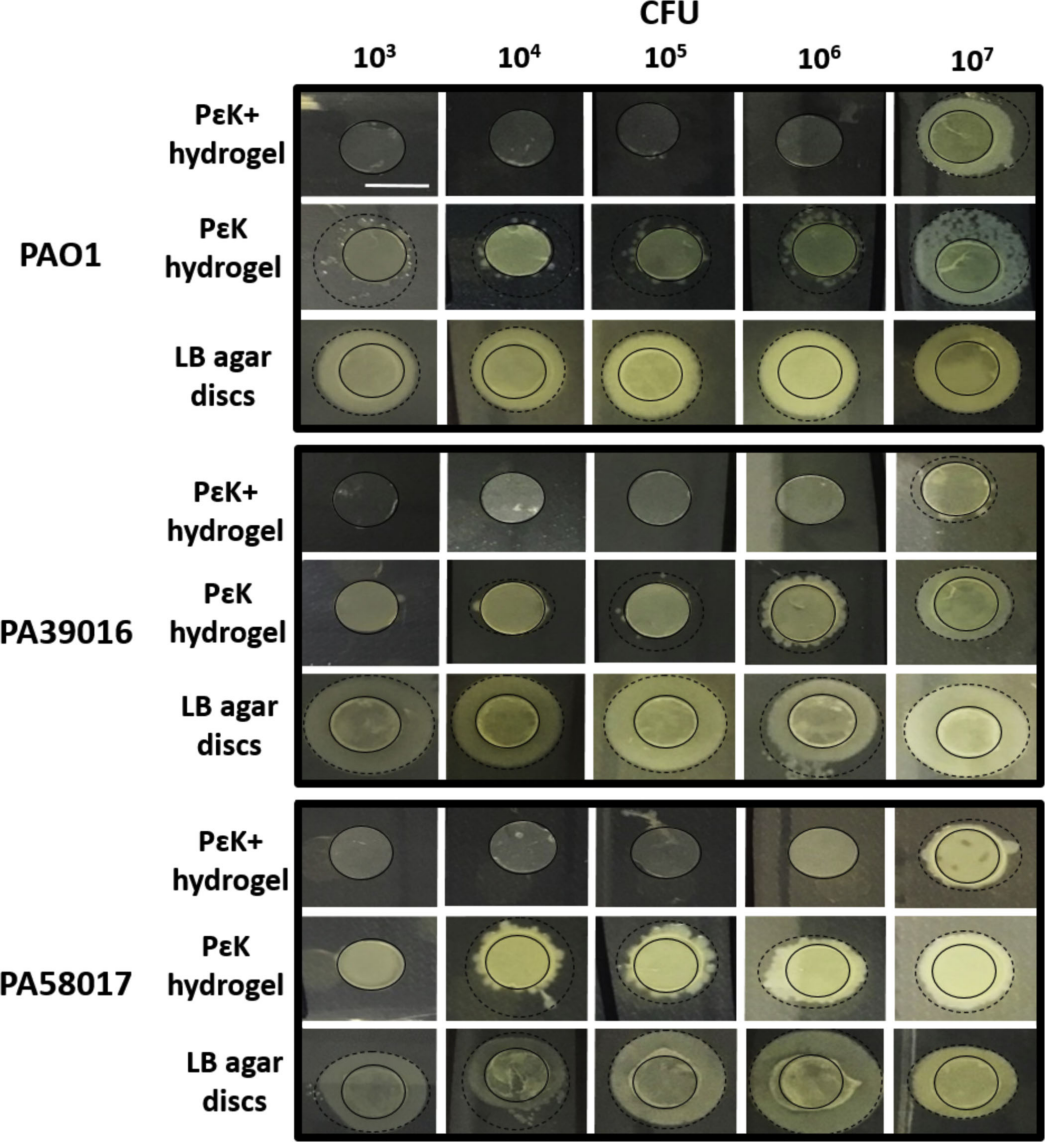
Reduction in *P* *aeruginosa* outgrowth from pɛK+ hydrogels, pɛK hydrogels and LB agar discs. PɛK+ hydrogels, pԑK hydrogel, and LB agar discs were incubated overnight in *P* *aeruginosa* (PAO1, PA39016, and PA58017) at 10^3^, 10^4^, 10^5^, 10^6^, and 10^7^ CFU for 24 hours*,* removed from buffer and plated onto LB agar plates, incubated overnight at 37°C. The *solid* *black circle* demarks the edge of the hydrogels or LB agar disc, dashed line demarks the edge of the bacterial outgrowth. Scale bar = 6 mm.

To determine whether we had successfully decreased the bacterial load on the pɛK+ hydrogels below the detection limit for viable counting, we assessed the growth of each strain directly from hydrogels and LB agar discs after incubation for 24 hours. There were no culturable cells of any of the *P* *aeruginosa* strains from pɛK+ hydrogels at inocula up to 10^6^ CFU, whereas nonfunctionalized pɛK hydrogels and LB agar discs showed colony outgrowth at all inocula tested (Fig. 3).

### Antimicrobial Activity of pɛK+ Hydrogels Against *P aeruginosa* Compared With Commercial Hydrogel CL

**Figure 4.**
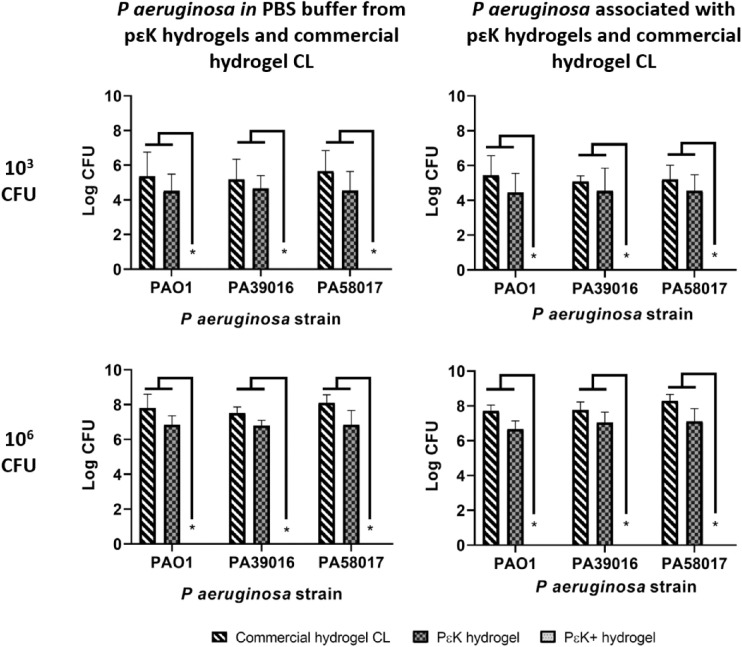
Antimicrobial activity of pԑK+ hydrogels, compared with pԑK hydrogels and commercial hydrogel CL against *P* *aeruginosa* isolates. Viable *P* *aeruginosa* in PBS buffer and associated with pԑK+ hydrogel, compared with pԑK hydrogel and commercial hydrogel CL. PԑK+ hydrogel, pԑK hydrogel, and commercial hydrogel CLs were inoculated with *P* *aeruginosa* (PAO1, PA39016, and PA58017) at 10^3^ and 10^6^ CFU for 24 hours. Viable bacterial counts were determined as CFU. Values represent mean of four independent experiments, error bars represent the standard deviation. *P < 0.05 using two-way ANOVA and post hoc Tukey's analysis.

Having determined that pɛK+ hydrogels possess antimicrobial activity against *P* *aeruginosa*, we investigated their antimicrobial effects compared with a commercial hydrogel CL at 10^3^ and 10^6^ CFU, after 24 hours incubation with different *P* *aeruginosa* strains. At 10^3^ CFU, pɛK+ hydrogels decreased the numbers of viable CFU from all strains of *P* *aeruginosa* in the PBS buffer or associated with pɛK+ hydrogels to below the level of detection, represented by more than 5 log and more than 4 log decreased, compared with the commercial hydrogel CL and nonfunctionalized pɛK hydrogel, respectively (Fig. 4). Using an inocula of 10^6^ CFU, *P* *aeruginosa* in the PBS buffer and associated with pɛK+ hydrogels were still less than the detection limit. For each of the *P* *aeruginosa* strains there were more than 7.5 and more than 6.0 log decreases in CFU in the PBS buffer and the pɛK+ hydrogels, compared with the commercial hydrogel CL and nonfunctionalized pɛK hydrogel, respectively. Nonfunctionalized pɛK hydrogels showed no significant log decreases compared with the commercial hydrogel CL.

### Antimicrobial Effects of pɛK+ Hydrogels Against *P aeruginosa* on Ex Vivo Corneas

**Figure 5.**
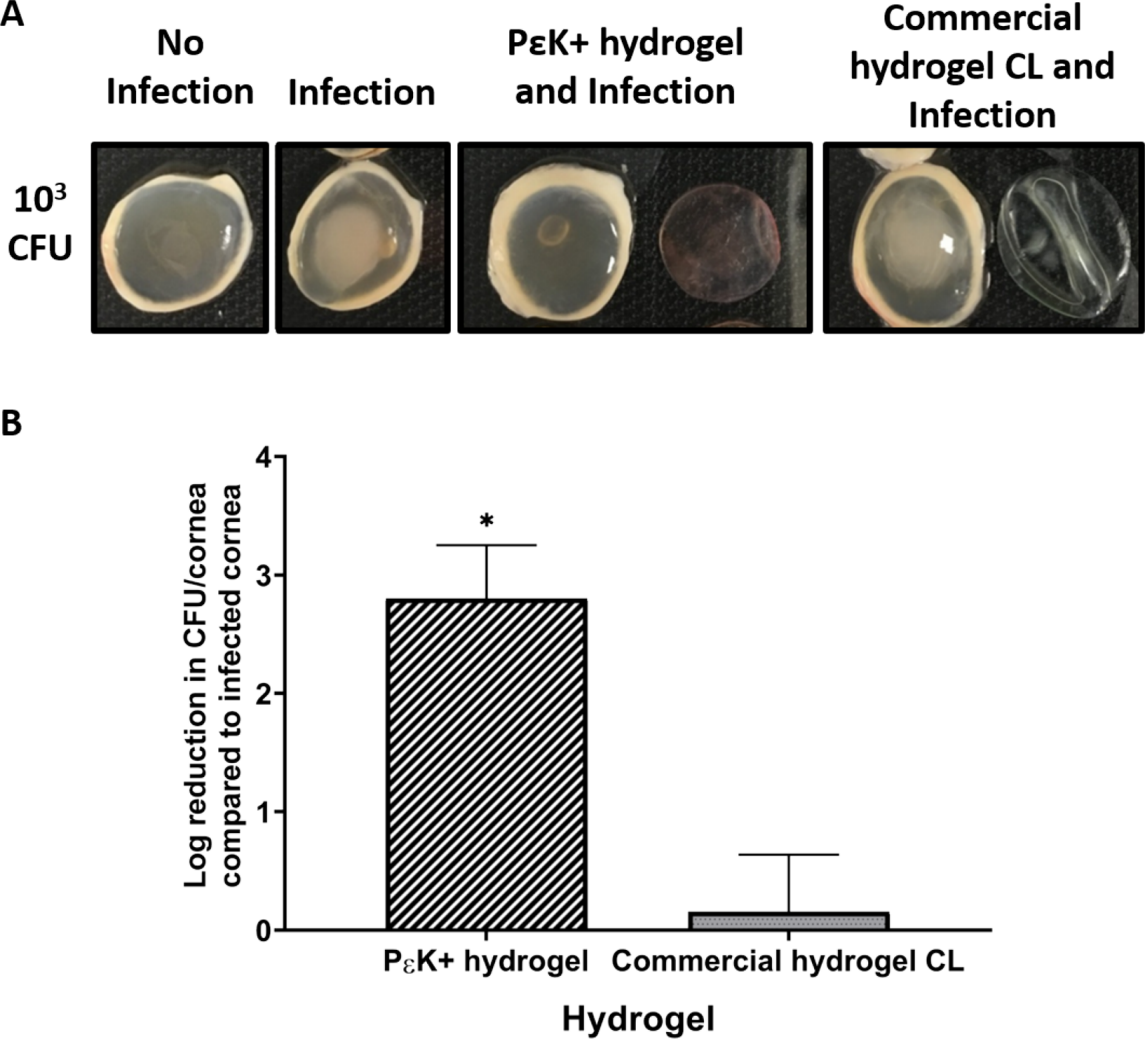
PɛK+ hydrogels prevent corneal haze and decrease CFU in cornea after PAO1 infection in an ex vivo porcine cornea infection model. (A) Growth of *P* *aeruginosa* PAO1 on porcine ex vivo corneas for 24 hours after infection with or without pԑK+ hydrogel or commercial hydrogel CL. Initial inocula size 10^3^ CFU/cornea. Reduction in corneal damage in presence of pԑK+ hydrogel compared with infected cornea and commercial hydrogel CL. (B) Reduction of *P* *aeruginosa* PAO1 retrieved from homogenized porcine ex vivo corneas infected with 10^3^ CFU, 24 hours after infection, with pԑK+ hydrogel or commercial hydrogel CL, compared with infected cornea. Viable bacterial counts were determined as CFU/cornea. Values represent mean, error bars represent the standard deviation, *n* = 4 independent experiments. *P < 0.05 versus infected cornea.

The antimicrobial effects of pɛK+ hydrogels and commercial hydrogel CL were investigated using an ex vivo porcine corneal infection model. Corneas infected with *P* *aeruginosa* PAO1 at 10^3^ CFU with pɛK+ hydrogels applied displayed decreased corneal haze and clouding at 24 hours, compared with infected corneas and corneas infected in the presence of the commercial hydrogel CL (Fig. 5A). Bacterial numbers of strain PAO1 associated with the corneas were quantified by CFUs; there was a 2.79 ± 0.45 log decrease in CFU in the presence of pɛK+ hydrogels, compared with the infected cornea. The commercial hydrogel CL showed a negligible 0.01 ± 0.41 log decrease in CFU compared with the infected corneas (Fig. 5B).

### Histologic Analysis of *P aeruginosa* on Ex Vivo Corneas

**Figure 6.**
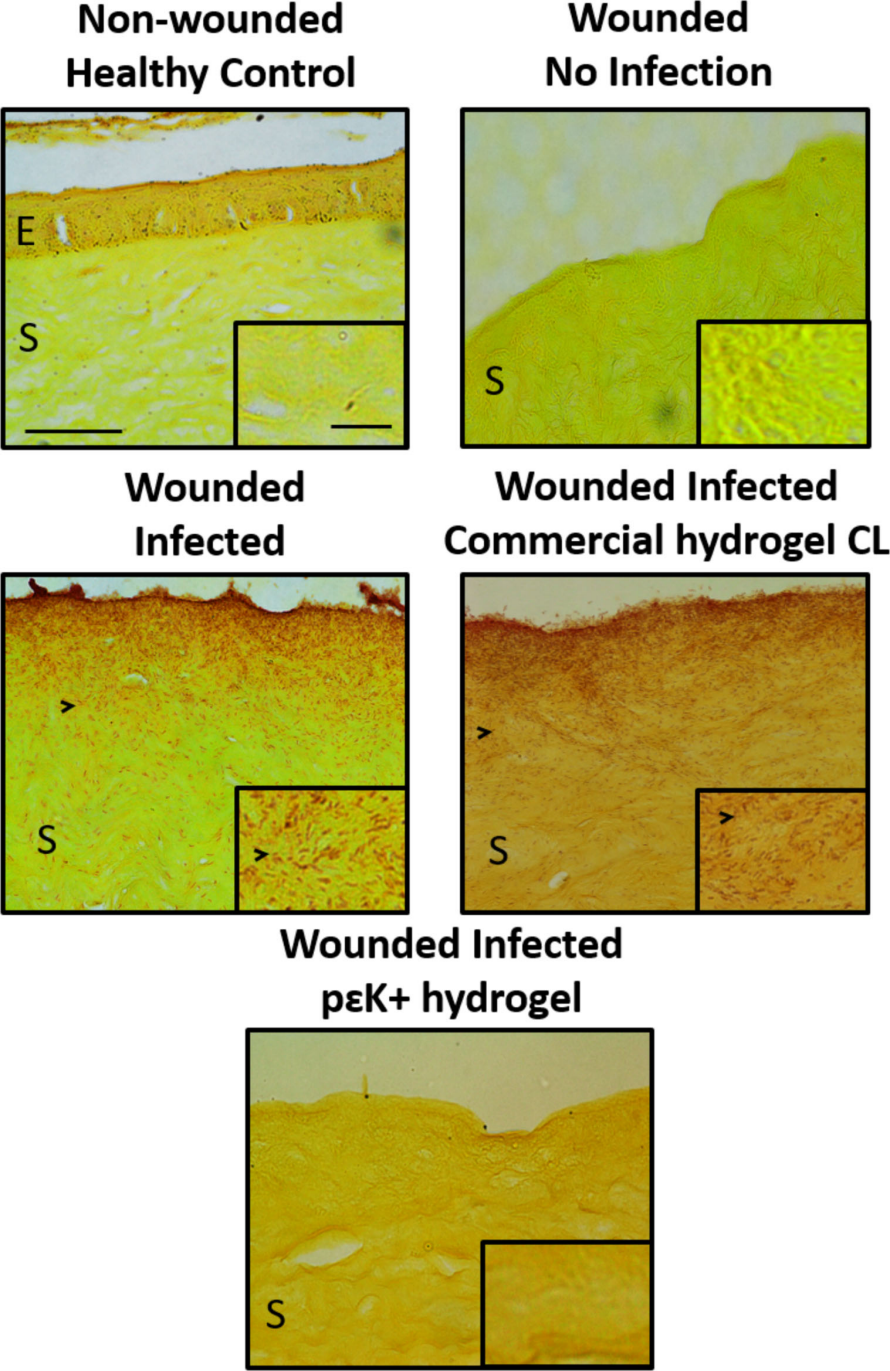
PɛK+ hydrogels prevent PAO1 infection in ex vivo porcine corneas. Histologic analysis of gram-stained corneal tissue sections from an ex vivo cornea infection model incubated with *P* *aeruginosa* strain PAO1 for 24 hours, with or without pԑK+ hydrogel or commercial hydrogel CL. Images show nonwounded healthy cornea showing intact epithelium (E) and stroma (S) and wounded cornea in the absence of infection with no epithelium present (tissue stained *yellow*). Wounded infected corneas with or without pԑK+ hydrogel or commercial hydrogel CL, bacteria stained *red* (labelled with *arrow heads*) throughout stromal tissue. Scale bar = 50 µm, insert scale bar = 10 µm.

The effects of pɛK+ hydrogels on the localization of strain PAO1 within ex vivo corneas was investigated using histologic analysis and compared with infected corneas or those with the commercial hydrogel CL. Control nonwounded and wounded corneas, showed the presence and absence of an intact epithelium, respectively, with no evidence of bacteria within the corneal stroma (Fig. 6). In contrast, strain PAO1 wounded infected corneas showed the presence of the bacteria stained red using Gram staining, localized intensely at the corneal surface and gradual invasion into the stroma. Corneas wounded and infected with strain PAO1 and incubated in the presence of the commercial hydrogel CL showed the presence of bacteria at the corneal surface and throughout the stroma, comparable with infected corneas. Corneas wounded and infected with strain PAO1 and incubated with pɛK+ hydrogel showed no evidence of bacteria directly under the hydrogel surface or throughout the stroma.

## Discussion

CLs are important and popular medical devices helping with visual and ocular surface restoration. They are, however, associated with an increased risk of MK, particularly that owing to *P* *aeruginosa.*^8^^,^^9^ This risk becomes particularly problematic when the integrity of the ocular surface is disturbed. Provision, therefore, of a CL with inherent antimicrobial properties that can be used as a bandage CL would be of benefit in decreasing the risks of MK and in providing protection of the ocular surface. In this study, we demonstrated that hydrogel functionalization with pɛK increased its antimicrobial properties via enhanced activity against laboratory and clinically relevant *exoU*^+^ and *exoS*^+^ keratitis strains of *P* *aeruginosa*, compared with the nonfunctionalized pɛK hydrogels and commercial hydrogel CL. This work supports the study from Gallagher et al., which demonstrated that pɛK functionalized hydrogels showed increased antimicrobial activity against laboratory strains of gram-positive *S* *aureus* and gram-negative *E* *coli.*^26^

PɛK+ hydrogels showed increased antimicrobial activity compared with nonfunctionalized pɛK hydrogels within the PBS buffer for all inocula and *P* *aeruginosa* strains; a scenario representing bacteria within the external environment of the CL and ocular surface. The effect of the inocula size upon pɛK and pɛK+ hydrogels was apparent at 4 hours, with higher inocula showing the lowest log decreases or no antimicrobial activity, possibly owing to the decreased ratio of molecules per bacteria. More important, we observed that pɛK+ hydrogels reduced viable CFU below the detection limit for all strains of *P* *aeruginosa* within the surrounding PBS buffer environment compared with the commercial hydrogel CL. In contrast, both nonfunctionalized pɛK hydrogels and commercial hydrogels showed bacterial growth of less than 1.5 log above the starting inocula. CLs used as therapeutic bandages are in contact with the cornea for longer than 4 hours, so the extent of the reductions at 24 hours of *P* *aeruginosa* with pɛK+ hydrogels is particularly encouraging for antimicrobial CL development.

CLs provide a potential surface for bacteria to colonize, proliferate, and infect the cornea, with adhesion being the initial step. Interestingly, *P* *aeruginosa* reportedly adheres at greater numbers than *S* *aureus* to CLs; however, different strains of *P* *aeruginosa* do not adhere differently.^34^^,^^35^ We obtained similar log decreases in CFU with pɛK+ hydrogels against all *P* *aeruginosa* strains for each inocula size and time point tested. Importantly, we observed antimicrobial activity against the highly virulent *exoU* isolate PA39016, isolated from a patient with prolonged healing time and resistance to antibiotics.^30^ Decreases in bacteria associated with pɛK+ hydrogels may prevent and retard any further growth or spreading onto the cornea. Further studies are required to investigate if there is biofilm formation or if any resistance evolves to pɛK+ hydrogels over time.

We assessed antimicrobial activity at 4 and 24 hours, representing logarithmic and stationary phases of bacterial growth, respectively, and this assessment was undertaken in a nutrient-limited PBS buffer to provide an environment representative of the ocular surface.^34^^,^^36^ Our data demonstrate that pɛK+ hydrogels have an effect on a range of inocula sizes. At 4 hours we observed that the largest inocula sizes were associated with the greatest adhesion to pɛK+ and nonfunctionalized pɛK hydrogels, consistent with other studies using alternative CLs.^34^ After 24 hours incubation, however, bacterial CFUs were decreased for all inocula sizes, indicating that the pɛK+ hydrogels are effective at providing antimicrobial activity against larger inocula sizes and preventing any further growth on the pɛK+ hydrogel for up to 24 hours. The infectious dose of pathogenic bacteria before colonization of the eye or CLs is unknown. Sweeney et al.^37^ reported approximately 10 to 30 CFU in a noninfectious environment after 13 nights wear of a soft, high water content ionic lens. We suspect that CLs and corneas are in contact with lower inocula sizes compared with those tested in our experiments. Our data would suggest pɛK+ hydrogels could prevent colonization and growth of bacteria on both the cornea and surrounding environment in the presence of a low inocula.

In this study, we demonstrated that pɛK+ hydrogels decreased the number of viable CFU in ex vivo porcine corneas, compared with infected corneas with and without a commercial hydrogel CL. We observed lower log reductions in bacterial numbers from pɛK+ hydrogels in ex vivo corneas, compared with in vitro experiments. PɛK+ hydrogel activity acts by contact and high bacterial numbers arising from growth on the cornea are likely to invade deeper into the stroma, limiting the antimicrobial effect of pɛK+ hydrogels. Ex vivo cornea models have limitations compared with in vivo models, such as a lack of tear fluid and the immune response. They do, however, provide an understanding of cellular and structural changes that occur in the cornea following an external insult such as a microbial infection and CL.^33^

Other studies have used different strategies to modify existing commercial CLs, which include both chemical and passive modifications using silver, free radicals, antimicrobial peptides, or nitric oxide–releasing polymers to either kill the bacteria or reduce adhesion with the CL surface.^38^^–^^40^ Although these strategies have their own advantages, a critically important advantage of pɛK hydrogels are their capacity to be functionalized owing to the free amine groups to exert increased antimicrobial activity. Although pɛK was used in this study, alternative agents can be attached similarly to engender additional antimicrobial activity or to improve healing of the ocular surface. Additional benefits of covalently binding additional pԑK to the free amine groups on the hydrogels is that the antimicrobial activity does not rely on the release of any antimicrobial agents via diffusion onto the ocular surface. Therapeutic effects of the pԑK hydrogel are therefore not decreased owing to the presence of corneal barriers such as increased tear fluid and blinking reflex.^26^

Previous studies have demonstrated the antimicrobial activity of pɛK+ hydrogels against laboratory strains of *S* *aureus* and *E* *coli*,^26^ and this study demonstrates antimicrobial activity against clinically relevant *P* *aeruginosa*, expanding its application as an antimicrobial CL. In summary, this study demonstrates the development of the pɛK+ hydrogels as antimicrobial CL that are effective against clinically relevant *P* *aeruginosa* strains could potentially lessen the risk of CL associated MK.

## Supplementary Material

Supplement 1

Supplement 2
