# Supplementary material for: Antimicrobial Activity of Poly-epsilon-lysine Peptide Hydrogels Against Pseudomonas aeruginosa
Source: Invest Ophthalmol Vis Sci. 2020 Aug 10;61(10):18. doi: 10.1167/iovs.61.10.18 (PMC7441358; doi:10.1167/iovs.61.10.18)
Supplement: Supplement 1 [file iovs-61-10-18_s001.pdf]

Table S1. Log reduction in *P. aeruginosa* (CFU) at 4 and 24 h in PBS buffer

PBS Buffer - 4 h

PA01 CFU	Log fold reduction pεk hydrogel to LB agar discs	Log fold reduction pεk+ hydrogel to pεk hydrogel	Log fold reduction pεk+ hydrogel to LB agar discs
10^3	3.03 (SD 0.93)*	3.77 (SD 0.52)*	6.75 (SD 0.60)*
10^4	3.00 (SD 0.20)*	4.18 (SD 0.25)*	7.17 (SD 0.23)*
10^5	3.11 (SD 0.98)*	3.55 (SD 1.56)*	6.65 (SD 1.47)*
10^6	2.46 (SD 1.06)*	2.94 (SD 1.83)*	5.39 (SD 1.06)*
10^7	2.07 (SD 1.84)	2.49 (SD 2.68)*	4.56 (SD 1.57)*

PA39016 CFU	Log fold reduction pεk hydrogel to LB agar discs	Log fold reduction pεk+ hydrogel to pεk hydrogel	Log fold reduction pεk+ hydrogel to LB agar discs
10^3	2.41 (SD 1.18)*	2.43 (SD 1.11)*	4.85 (SD 0.41)*
10^4	2.41 (SD 1.18)*	2.43 (SD 1.11)*	4.85 (SD 0.41)*
10^5	1.97 (SD 0.96)*	2.39 (SD 0.69)*	4.36 (SD 0.45)*
10^6	1.26 (SD 0.41)	2.41 (SD 0.82)*	3.67 (SD 0.93)*
10^7	1.33 (SD 1.10)	1.63 (SD 1.50)*	2.97 (SD 1.15)*

PA58017 CFU	Log fold reduction pεk hydrogel to LB agar discs	Log fold reduction pεk+ hydrogel to pεk hydrogel	Log fold reduction pεk+ hydrogel to LB agar discs
10^3	2.46 (SD 1.42)*	3.57 (SD 0.60)*	6.07 (SD 1.57)*
10^4	2.82 (SD 0.77)*	4.18 (SD 1.11)*	7.00 (SD 1.22)*
10^5	2.58 (SD 0.46)*	3.23 (SD 2.56)*	5.82 (SD2.19)*
10^6	2.26 (SD 1.36)	2.42 (SD 0.94)*	4.69 (SD 1.06)*
10^7	-0.34 (SD 0.30)	3.95 (SD 0.87)*	3.60 (SD 1.00)*

PBS Buffer - 24 h

PA01 CFU	Log fold reduction pεk hydrogel to LB agar discs	Log fold reduction pεk+ hydrogel to pεk hydrogel	Log fold reduction pεk+ hydrogel to LB agar discs
10^3	4.69 (SD 0.96)*	5.34 (SD 0.87)*	10.03 (SD 0.40)*
10^4	3.98 (SD 1.80)*	6.42 (SD 1.09)*	10.40 (SD 1.65)*
10^5	2.85 (SD 1.14)*	7.84 (SD 0.76)*	10.69 (SD 1.17)*
10^6	1.36 (SD 1.14)*	9.27 (SD 1.17)*	10.62 (SD 1.21)*
10^7	0.58 (SD 0.79)	9.06 (SD 1.91)*	9.64 (SD 2.08)*

PA39016 CFU	Log fold reduction pεk hydrogel to LB agar discs	Log fold reduction pεk+ hydrogel to pεk hydrogel	Log fold reduction pεk+ hydrogel to LB agar discs
10^3	5.66 (SD 1.49)	4.53 (SD 0.85)*	10.20 (SD 0.75)*
10^4	5.90 (SD 1.16)*	4.74 (SD 1.29)*	10.64 (SD 0.34)*
10^5	5.00 (SD 0.96)*	5.64 (SD 1.11)*	10.64 (SD 0.27)*
10^6	3.83 (SD 1.16)*	7.04 (SD 1.21)*	10.87 (SD 0.24)*
10^7	2.64 (SD 1.37)*	7.94 (SD 0.53)*	10.57 (SD 0.99)*

PA58017 CFU	Log fold reduction pεk hydrogel to LB agar discs	Log fold reduction pεk+ hydrogel to pεk hydrogel	Log fold reduction pεk+ hydrogel to LB agar discs
10^3	4.41 (SD 1.22)*	5.47 (SD 1.24)*	9.88 (SD 0.27)*
10^4	4.29 (SD 1.71)*	6.12 (SD 1.23)*	10.34 (SD 0.93)*
10^5	3.39 (SD1.05)*	6.12 (SD 0.75)*	10.62 (SD 0.81)*
10^6	2.64 (SD 0.90)*	8.46 (SD 1.15)*	11.10 (SD 0.76)*
10^7	1.03 (SD 0.88)	7.62 (SD 2.24)*	8.71 (SD 2.84)*

Log reduction comparisons of *P. aeruginosa* viable counts in PBS buffer following incubation with pεK+ hydrogel, pεK hydrogel and LB agar discs at 4 and 24 h for inocula sizes of 10^3, 10^4, 10^5, 10^6 and 10^7 CFU. Symbol * indicates significantly different $P<0.05$, two-way ANOVA and post hoc Tukey's analysis.
