# Supplementary material for: Antimicrobial Activity of Poly-epsilon-lysine Peptide Hydrogels Against Pseudomonas aeruginosa
Source: Invest Ophthalmol Vis Sci. 2020 Aug 10;61(10):18. doi: 10.1167/iovs.61.10.18 (PMC7441358; doi:10.1167/iovs.61.10.18)
Supplement: Supplement 2 [file iovs-61-10-18_s002.pdf]

Table S2. Log reduction in *P. aeruginosa* (CFU) at 4 and 24 h associated with pεK hydrogels or LB agar discs

PεK hydrogels and LB agar discs - 4 h

PA01 CFU	Log fold reduction pεk hydrogel to LB agar discs	Log fold reduction pεk+ hydrogel to pεk hydrogel	Log fold reduction pεk+ hydrogel to LB agar discs
10^3	2.19 (SD 0.60)*	3.58 (SD 0.54)*	5.77 (SD 0.84)*
10^4	2.17 (SD 0.88)*	3.90 (SD 0.94)*	6.07 (SD 1.28)*
10^5	2.62 (SD 1.04)*	3.67 (SD 1.51)*	6.29 (SD 1.33)*
10^6	2.15 (SD 1.31)*	2.50 (SD 0.82)*	4.64 (SD 0.77)*
10^7	0.95 (SD 1.14)	2.28 (SD 1.36)*	3.24 (SD 1.18)*

PA39016 CFU	Log fold reduction pεk hydrogel to LB agar discs	Log fold reduction pεk+ hydrogel to pεk	Log fold reduction pεk+ hydrogel to LB agar discs
10^3	2.35 (SD 0.61)*	2.95 (SD 0.86)*	5.30 (SD 1.03)*
10^4	2.49 (SD 1.18)*	2.57 (SD 0.95)*	5.06 (SD 1.69)*
10^5	1.85 (SD 0.98)*	3.35 (SD 1.39)*	5.19 (SD 2.12)*
10^6	1.32 (SD 0.90)	2.25 (SD 0.45)*	3.57 (SD 1.0)*
10^7	0.89 (SD 1.71)	2.15 (SD 1.09)*	3.04 (SD 0.91)*

PA58017 CFU	Log fold reduction pεk hydrogel to LB agar discs	Log fold reduction pεk+ hydrogel to pεk hydrogel	Log fold reduction pεk+ hydrogel to LB agar discs
10^3	2.34 (SD 1.25)*	4.34 (SD 0.35)*	6.67 (SD 0.91)*
10^4	1.87 (SD 1.16)*	4.67 (SD 1.80)*	6.56 (SD 1.55)*
10^5	2.14 (SD 1.05)*	4.70 (SD 1.64)*	6.27 (SD 1.46)*
10^6	1.59 (SD 0.75)	3.00 (SD 0.38)*	4.58 (SD 0.89)*
10^7	0.78 (SD 0.89)	2.85 (SD 1.35)*	3.60 (SD 1.20)*

PεK hydrogels and LB agar disc - 24 h

PA01 CFU	Log fold reduction pεk hydrogel to LB agar discs	Log fold reduction pεk+ hydrogel to pεk hydrogel	Log fold reduction pεk+ hydrogel to LB agar discs
10^3	4.81 (SD 1.67)*	4.75 (SD 1.64)*	9.58 (0.64)*
10^4	3.54 (SD 1.53)*	5.98 (SD 1.30)*	9.51 (SD 1.47)*
10^5	1.96 (SD 0.79)*	7.85 (SD 1.22)*	9.79 (SD 1.54)*
10^6	1.22 (SD 0.81)	8.76 (SD 1.89)*	10.00 (SD 1.42)*
10^7	0.78 (SD 0.82)	8.42 (SD 1.67)*	9.20 (SD 1.79)*

PA39016 CFU	Log fold reduction pεk hydrogel to LB agar discs	Log fold reduction pεk+ hydrogel to pεk hydrogel	Log fold reduction pεk+ hydrogel to LB agar discs
10^3	4.68 (SD 1.51)*	4.64 (SD 1.18)*	9.32 (SD 0.97)*
10^4	4.31 (SD 1.45)*	5.58 (SD 1.58)*	9.89 (SD 0.97)*
10^5	3.47 (SD 1.84)*	6.77 (SD 2.10)*	10.23 (SD 0.68)*
10^6	2.25 (SD 1.26)*	7.56 (SD 1.88)*	9.81 (SD 1.37)*
10^7	0.83 (SD 0.99)	9.50 (SD 1.35)*	10.32 (SD 1.00)*

PA58017 CFU	Log fold reduction pεk hydrogel to LB agar discs	Log fold reduction pεk+ hydrogel to pεk hydrogel	Log fold reduction pεk+ hydrogel to LB agar discs
10^3	3.66 (SD 1.03)*	5.31 (SD 1.35)*	8.97 (SD 1.16)*
10^4	3.21 (SD 1.80)*	6.52 (SD 1.42)*	9.73 (SD 1.39)*
10^5	2.70 (SD 1.16)*	7.30 (SD 1.18)*	10.00 (SD 1.31)*
10^6	1.54 (SD 1.65)*	7.78 (SD 1.02)*	9.32 (SD 1.31)*
10^7	0.57 (SD 1.47)	7.34 (SD 0.87)*	7.91 (SD 0.87)*

Log reduction comparisons of *P. aeruginosa* viable counts associated with pεK+ hydrogel, pεK hydrogel and LB agar discs at 4 and 24 h for inocula sizes of 10^3, 10^4, 10^5, 10^6 and 10^7 CFU. Symbol * indicates significantly different $P<0.05$, two-way ANOVA and post hoc Tukey's analysis.
